# Noncontact Longitudinal Respiratory Rate Measurements in Healthy Adults Using Radar-Based Sleep Monitor (Somnofy): Validation Study

**DOI:** 10.2196/36618

**Published:** 2022-08-12

**Authors:** Ståle Toften, Jonas T Kjellstadli, Ole Kristian Forstrønen Thu, Ole-Johan Ellingsen

**Affiliations:** 1 Department of Data Science and Research VitalThings AS Tønsberg Norway; 2 VitalThings AS Tønsberg Norway; 3 Department of Anesthesiology and Intensive Care Medicine Levanger Hospital Nord-Trøndelag Hospital Trust Levanger Norway

**Keywords:** noncontact, monitoring, radar technology, respiratory rate, Somnofy, validation

## Abstract

**Background:**

Respiratory rate (RR) is arguably the most important vital sign to detect clinical deterioration. Change in RR can also, for example, be associated with the onset of different diseases, opioid overdoses, intense workouts, or mood. However, unlike for most other vital parameters, an easy and accurate measuring method is lacking.

**Objective:**

This study aims to validate the radar-based sleep monitor, Somnofy, for measuring RRs and investigate whether events affecting RR can be detected from personalized baselines calculated from nightly averages.

**Methods:**

First, RRs from Somnofy for 37 healthy adults during full nights of sleep were extensively validated against respiratory inductance plethysmography. Then, the night-to-night consistency of a proposed filtered average RR was analyzed for 6 healthy participants in a pilot study in which they used Somnofy at home for 3 months.

**Results:**

Somnofy measured RR 84% of the time, with mean absolute error of 0.18 (SD 0.05) respirations per minute, and Bland-Altman 95% limits of agreement adjusted for repeated measurements ranged from –0.99 to 0.85. The accuracy and coverage were substantially higher in deep and light sleep than in rapid eye movement sleep and wake. The results were independent of age, sex, and BMI, but dependent on supine sleeping position for some radar orientations. For nightly filtered averages, the 95% limits of agreement ranged from −0.07 to −0.04 respirations per minute. In the longitudinal part of the study, the nightly average was consistent from night to night, and all substantial deviations coincided with self-reported illnesses.

**Conclusions:**

RRs from Somnofy were more accurate than those from any other alternative method suitable for longitudinal measurements. Moreover, the nightly averages were consistent from night to night. Thus, several factors affecting RR should be detectable as anomalies from personalized baselines, enabling a range of applications. More studies are necessary to investigate its potential in children and older adults or in a clinical setting.

## Introduction

### Background

Respiratory rate (RR) is arguably the most valuable parameter to detect clinical deterioration in hospital wards [1-3], and it is an important measure of health and wellness. Substantial change in RR can be associated with lower respiratory tract infections [4], fever [5,6], acute asthma [7], acute brain damage [8], opioid overdose [9], or exacerbation of chronic obstructive pulmonary disease (COPD) [10,11]. Other factors such as intense workouts [12], emotions or anxiety [13], and menstrual cycle [14] have also been shown to affect RR. A solution that can automatically, conveniently, and continuously monitor RR can have a range of applications.

In recent years, many new methods and devices have been developed to measure RR [15], but an accurate and simple method is still lacking [16]. Capnography is sometimes regarded as the gold standard [15,17], but hospitals still use manual counting of breaths [18], even though chest patches and under-the-mattress sensors are also available [19]. Chest patches derive RR from electrical cardiography by analyzing respiration-induced modulations on the heart signals, a technique also used in photoplethysmography in consumer wearables [20]. Studies on sleep use wearables such as thermistors, nasal pressure, and respiratory inductance plethysmography (RIP) to measure respiration. Although some of these technologies are accurate, they are unfortunately not suitable for longitudinal studies. It would be preferable for such a device to be noncontact and mobile and not need recharging or maintenance. As RRs vary during the day, measurements are often performed when the person is resting to obtain consistent measurements. To increase consistency, it can be advantageous to measure during nighttime, when the person sleeps and is unable to affect the measurements intentionally or unintentionally. Nocturnal RRs have also been shown to be an independent predictor of long-term mortality (RRs >16 respirations [breaths] per minute [RPM]) [21], and nocturnal hyperpnea is shown to be an indicator of periodic limb movement disorders [22]. Different under-the-mattress sensors have been investigated for this purpose [23-25], but radar technology is also an alternative.

Radar technology has been extensively studied for measuring RR [26-29] and even for detecting apnea [26,30-32]. However, most studies have measured RR only during optimized conditions where the participant is asked to sit or lie still [26] or during natural movements or sleep, but only for short periods [28,29]. A recent study validated RRs during full nights of sleep in both healthy individuals and patients with sleep apnea, but their study included only 6 healthy participants and the precision decreased significantly for participants with sleep apnea [27]. Moreover, their study did not analyze factors that may affect precision, such as body position (prone, supine, and side), sleep stage, or BMI or for how much of the night the radar was able to measure RR. There is still a need for more validation of radar technology for continuous monitoring during sleep. Furthermore, most studies dealing with RRs compare spot measurements with aggregate statistics that combine measurements from different people [33,34], even though there are large variations between individuals within the normal range [35,36]. Thus, the technology will be more useful if it can also be used to establish meaningful personalized baselines. The development and use of such baselines must be carefully considered, as RRs vary extensively even throughout the night. Thus, a person can easily seem ill in one moment and healthy in the next, when using standard spot measurements. For the technology to be able to reliably detect events affecting RR, it is vital that both the normal variations and measurement error for RR are significantly smaller than the effect of the event.

### Objectives

The aim of this study was to investigate whether a commercially available radar-based sleep monitor, Somnofy (VitalThings), can be used as a longitudinal RR monitor. The first objective was to extensively benchmark RR measurements from Somnofy against those derived from RIP. This objective included both instantaneous measurements and filtered nightly averages, which are proposed as robust metrics in longitudinal monitoring of RR. The second objective was to analyze the night-to-night consistency of this nightly average in a pilot study over 3 months and investigate whether it can be possible to reliably detect events affecting RR as deviations from personalized baselines with this type of technology.

## Methods

### Participants and Data Sample

For the first part of the study, 55 volunteers from Norway were recruited to sleep 1 night at a sleep laboratory. The participants were recruited directly or through social media. The inclusion criterion was healthy adults aged >18 years. In total, 33% (18/55) of the individuals were later removed from the data set. Of these 18 individuals, 15 (83%) were excluded owing to indications of sleep-related disorders that can influence RR (sleep apnea and periodic limb movement disorder), whereas 3 (17%) were excluded owing to initial recording problems (the recordings lacked >2 hours of data). Thus, the first data set contained data from 1 night of sleep from 67% (37/55) of the healthy adults (21/37, 57% were women). The average age was 32.6 (SD 10.6) years, and they had an average BMI of 23.3 (SD 2.9) kg/m^2^.

For the second part of the study, 6 Norwegian individuals (aged 11-81 years; n=2, 33% were women) were recruited to use Somnofy at home for 3 months. The inclusion criterion was the participants to be in good health, which meant that they did not have any disease that increased the possibility for hospitalization, causing discontinuity in the measurements.

### Ethical Considerations

As this was not a clinical study and included only healthy participants, it was exempted from review in accordance with the Norwegian National Research Ethics Committee (reference number: "2019/995 A", June, 2019). Written informed consent was obtained from all participants in accordance with the principles embodied in the Declaration of Helsinki. All methods were performed in accordance with relevant guidelines and regulations.

### Procedure

The participants in the first phase slept 1 night at the sleep laboratory in the Colosseum Clinic in Oslo, Norway. They were not allowed to consume alcohol or other drugs 48 hours before the assessments and could not smoke during the assessments. Full polysomnography (PSG) was performed to detect possible sleep disorders. The PSG data were also used to derive RRs, which were later compared with the output from Somnofy. In total, 2 Somnofy units recorded each night. One unit was placed on a nightstand to the left of the participant and the other unit was placed on the wall above the participant’s head. Both units were aimed at the participant’s chest. Overall, 4 participants lacked data from one of the sensors. Consequently, data from only 1 randomly selected sensor were used per participant (nightstand: 20/37, 54% and wall: 17/37, 46%). However, for the analyses specifically investigating the difference between the 2 sensor locations, both sensors were used, and the 4 participants with only 1 sensor location were dropped.

In the second part of the study, the participants were each given 1 Somnofy unit to take home, and they were instructed to place it on their nightstand. The adult participants (3/6, 50%) shared beds with their spouses. For these participants, the Somnofy distance parameter was set to a distance at the midpoint between the 2 individuals’ thoraces in a normal sleeping position. No problems were detected with this setup, and no data were removed owing to disruption by the spouse. All participants were encouraged to live normally. During the study, 67% (4/6) of the participants experienced periods of self-reported illness. They did not recall the exact start or end times of these illnesses. As no illness occurred in the first half of the period, it was used to calculate personalized RR baselines for all participants (n=40 nights). Baselines were calculated as the average RR over the period, and 95% CIs were calculated as the baseline–1.96 × SD to baseline+1.96 × SD.

### Somnofy

Somnofy (version 0.7; VitalThings) was used in this study. Somnofy uses an impulse radio ultrawideband radar with an average sampling rate of 23.8 GHz, which, through configuration, is sampled into a 3-m–long frame of 5-cm bins updated with a frequency of approximately 17 Hz. Somnofy measures humans by emitting signals that are reflected by the human body. If the body moves, it will affect the signals that are returned to the radar. The RR is further derived by using the Doppler effect and signal processing techniques, primarily the Fast Fourier transform (FT), to analyze periodic movements caused by the chest wall. The Fast FT is calculated every second for the last 20 seconds of the data, using a Hann window and 19-second overlap. Artifacts and harmonics are automatically removed by Somnofy; therefore, it provides only estimates that it is confident in. In this study, Somnofy was configured to provide RRs between 8 and 30 RPM. Movement is derived by analyzing the changes in the received radar signal over the last 6 seconds. The operating frequency enables the radar signal to travel through bedsheets and clothes before being reflected on the human body. More information on the principles of radar technology is available in previous studies [26].

Somnofy also calculates the nightly average RR. When calculating nightly averages, it is not necessary to use every instantaneous RR throughout the night. Using only selected measurements and filtering outliers can increase the night-to-night consistency, because the average does not depend on, for example, the amount of movement during the night. Therefore, Somnofy considers only periods without movement and rapid eye movement (REM) sleep, where RR tends to vary, when calculating nightly averages. In addition, outliers defined as >0.675 SD away from the mean are disregarded.

Somnofy is certified according to the Federal Communication Commission and “Conformité Européene” and harmless to human beings. Somnofy is installed by simply placing the unit on a nightstand or mounting it on a wall. Somnofy measures RR for 1 person and can do so despite the presence of 2 individuals in a bed, for which it measures only the nearest person if the person lies 5 cm closer to the radar than the other person. However, when 2 individuals are sharing a bed, the distance parameter in Somnofy should be set to a distance between the 2 individuals’ thoraces to prevent the unit from starting to measure the other person when the intended participant exits the bed. Somnofy also collects additional information about the sleeping environment and scores sleep stages (accuracy to detect sleep=0.97 and accuracy to detect wake=0.72 in epoch-by-epoch analyses) [37]. For more details about Somnofy, refer to the validation study on sleep stage classification [37]. Currently, Somnofy is not a Food and Drug Administration–approved medical device.

### PSG Recordings

PSG was performed using SOMNOscreen plus (SOMNOmedics) by sleep specialists following the guidelines of the American Academy of Sleep Medicine [38]. RRs were derived from 32 Hz RIP using the short-time FT (STFT) as implemented by the Python library *SciPy* (version 1.4.1). The STFT was calculated with a 20-second Hann window and a 19-second overlap, providing 1 measurement per second. For minimal noise, the RIP belt (thorax or abdomen) with the highest signal quality was used for each 20-second window to derive RR.

However, the RRs derived from RIP were still noisy when the input data quality was low. To remove this noise, all measurements that were more than double or less than half of the measurement in the previous second were disregarded. The RRs were filtered further by removing outliers, which were defined as measurements >1.96 SDs away from the mean of a 15-minute interval around the measurement.

Nightly average RRs for RIP were calculated using the same time stamps as those used by Somnofy. To synchronize the clocks in Somnofy and PSG, the cross-correlation between movement from Somnofy and PSG was maximized. Time in bed was defined as the time from lights out to lights on.

### Statistical Analysis

As RRs were measured continuously during the night, the Bland-Altman method [39] was chosen as the main statistical tool to validate Somnofy against RIP [40]. In contrast to techniques that predefine an acceptable error margin, the Bland-Altman limit of agreement can be considered across several applications, as the difference is quantified. For instantaneous measurements, the Bland-Altman limits of agreement were calculated per night, and on the combined data set, adjusting for multiple measurements per participant [41]. In addition, the mean absolute error (MAE) was used to measure the absolute deviance, the coefficient of determination (*R*^2^) was calculated to measure the correlation between RIP and Somnofy during the night, and coverage (percentage of the time Somnofy provided measurements regardless of whether RIP provided measurements) and gap (longest time that passed without a Somnofy measurement) were analyzed to investigate the reliability and robustness of the device.

MAE and coverage for instantaneous RRs were analyzed across age, sex, BMI, sensor location, and sleeping position for significant differences. The null hypothesis was that there was no difference with α set to .05. Age (young adult or adult), sex (male or female), and BMI (normal weight or overweight) were analyzed using a 2-tailed, 2-sample, unpaired *t* test, as the sample sizes were <30. To avoid bias toward individual participants, analyses were performed on average values for each night, disregarding waking periods. Calculations were performed using Python (version 3.6.8) and the *SciPy* (version 1.4.1) library.

From the radars’ point of view, sleeping position depended on sensor location. A total of 8 different position parameters were established as combinations of the four sleeping positions (supine, prone, left, and right) and the two sensor locations (nightstand and wall). For each night, combinations with <300 measurements were disregarded. Consequently, not all combinations were available for every night, because not all the participants slept in every sleeping position. As the data set was paired, had >2 levels, and was unbalanced, a linear mixed effects model was chosen to analyze statistical significance. For these analyses, only measurements taken during Somnofy-defined sleep were used. The output model was analyzed using the Tukey method to investigate individual pairwise relationships. The analyses were performed in R (version 3.6.3), using the *“lme4”* (version 1.1-23) and *“multcomp”* (version 1.4-13) packages.

## Results

### Data Statistics

**Figure 1:**
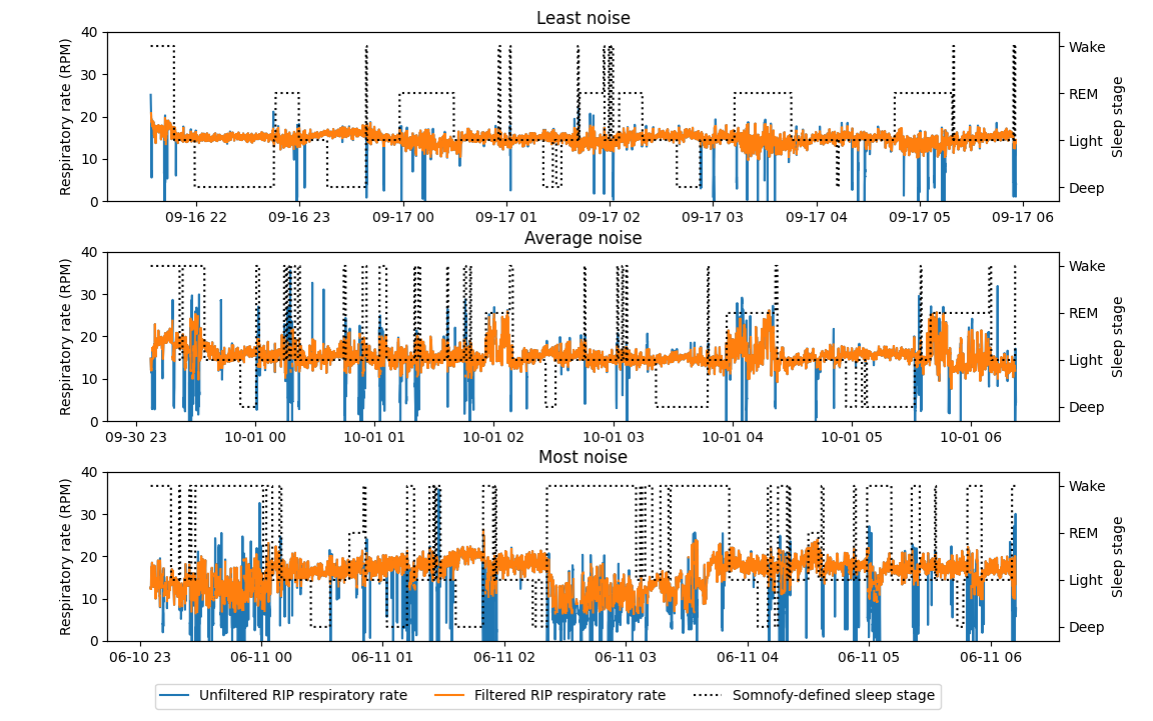
Nights with the least, average, and most noise removed from respiratory rates derived from respiratory inductance plethysmography (RIP). The respiratory rates as respirations per minute (RPM) are displayed on the y-axis, and the date and time (mm-dd HH) are displayed on the x-axis. The filter removes obvious outliers in the respiratory rate without removing normal variations during wake and rapid eye movement (REM) sleep.

Table 1 shows the age, sex, and BMI distribution of the participants in the validation part of the study, and Table 2 shows the relevant sleep and respiratory parameters. On average, the participants spent 8 (SD 0.7) hours in bed, with PSG-defined sleep efficiency of 85.3% (SD 8.3%). The average RIP RR ranged from 11 to 21.4 RPM. On average, 5.8% (SD 1.9%) of the data were removed per night owing to filtering of RIP noise. Of the 61,775 data points removed, most data were removed from PSG-defined wake (n=31,063, 50.28%), light sleep (n=19,137, 30.98%), and REM sleep (n=7,561, 12.24%). Figure 1 displays the noise filtering for RIP for the nights with the least, average, and most noise. The filter removed most outliers but did not remove the natural variations in RR that occur during wake and REM sleep. While removing time stamps with PSG artifacts, 3.42% (30,294/886,512) of the Somnofy data were removed.

**Table 1:** Age, sex, and BMI of the participants in the validation study (N=37).

Categories	Participants, n (%)	Female participants, n (%)	Age (years), mean (SD); range	BMI (kg/m^2^), mean (SD); range
All	37 (100)	21 (57)	32.6 (10.6); 20-62	23.3 (2.9); 18.5-28.7
Normal weight^a^	25 (68)	15 (41)	31.8 (10.7); 22-62	21.7 (2); 18.5-24.7
Overweight^b^	12 (32)	6 (16)	34.4 (10.8); 20-55	26.6 (1.1); 25.2-28.7
Young adult^c^	22 (59)	13 (35)	25.6 (2.6); 20-29	22.3 (2.9); 18.5-28.7
Adult^d^	15 (41)	8 (22)	42.9 (9.6); 31-62	24.6 (2.3); 20.8-27.8

^a^18.5≤BMI<25.

^b^BMI≥25.

^c^Aged <30 years.

^d^Aged ≥30 years.

**Table 2:** Sleep and respiratory parameters for the validation study (N=37).

Parameters	Values, mean (SD)	Values, range
PSG^a^—time in bed (hours)	8 (0.7)	6.8-10
PSG—sleep efficiency (%)	85.3 (8.3)	58.9-95.4
PSG—wake after sleep onset (minutes)	44.9 (33.8)	4-143.9
Somnofy—total sleep time (hours)	6.9 (0.9)	4.1-8.2
RIP^b^—noise removed (%)	5.8 (1.9)	3.1-12.5
RIP—average respiratory rate (RPM^c^)	15.5 (2.1)	11-21.4
AHI^d^	1.1 (1.1)	0-3.8
PLMI^e^	1.3 (2.8)	0-13.8
ArI^f^	9.2 (3)	2.6-18.29

^a^PSG: polysomnography.

^b^RIP: respiratory inductance plethysmography.

^c^RPM: respirations per minute.

^d^AHI: apneas and hypopneas per hour of sleep.

^e^PLMI: periodic limb movements per hour of sleep.

^f^ArI: arousals per hour of sleep.

### Instantaneous RR

**Figure 2:**
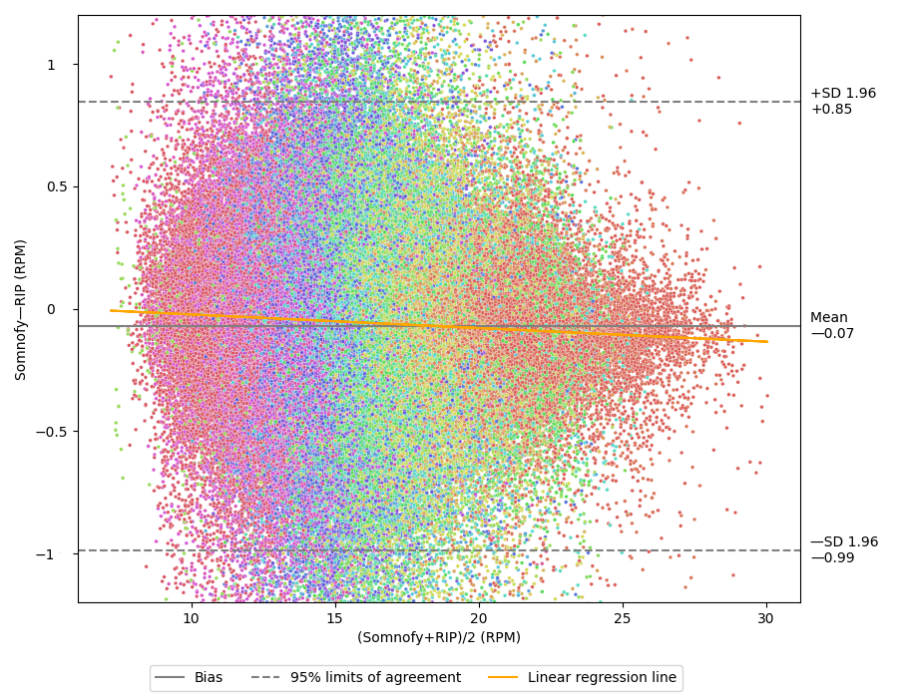
Bland-Altman plot for instantaneous respiratory rates. The y-axis displays the disagreement between Somnofy and respiratory inductance plethysmography (RIP; N=871,072), whereas the x-axis displays the average of Somnofy and RIP measurements. All values are presented as respirations per minute (RPM). Measurements from the same night are visualized with the same color. For overlapping measurements, the top measurements were picked randomly. The y-axis is limited to between –1.2 and 1.2.

The results of the measurements of instantaneous RR are displayed in Table 3. During time in bed, Somnofy managed to measure RR 84% (SD 6%) of the time (coverage) and the MAE of these measurements was 0.18 (SD 0.05) RPM compared with RIP. On average, the 95% limits of agreement with RIP ranged from −0.94 (SD 0.35) to 0.80 (SD 0.32) RPM, with bias of −0.07 (SD 0.02) RPM. After adjusting for repeated measurements, the limits of agreement on the whole data set ranged from −0.99 to 0.85. Figure 2 shows the Bland-Altman plot for this scenario. The orange regression line (slope=−0.0057; *R*^2^=0.0059) indicates that Somnofy tends to underestimate RR compared with RIP and that Somnofy underestimates more for high RRs. During Somnofy-defined sleep, the coverage was 90% (SD 3.7%) and the limits of agreement ranged from −0.83 (SD 0.28) to 0.69 (SD 0.25), on average. In particular, the worst nights were improved by removing wake data, indicating that these nights had high amount of wake, which was difficult for Somnofy to measure.

Table 4 shows the coverage and accuracy across the different Somnofy-defined sleep stages. Somnofy was most accurate during deep sleep (non-REM 3) and light sleep (non-REM 1 or non-REM 2), whereas accuracy and coverage were substantially lower during wake and REM sleep than during other sleep stages. The results across PSG-defined sleep stages were similar (Multimedia Appendix 1).

**Table 3:** Results for instantaneous respiratory rate (N=37).

Parameters	During time in bed, mean (SD); range	During Somnofy-defined sleep, mean (SD); range
Number of measurements^a^ (1000s)	28.66 (2.66); 24.48 to 36.07	24.69 (3.26); 14.88 to 29.67
Coverage^b^ (%)	83.5 (6); 69 to 93.3	89.5 (3.7); 80.2 to 97.3
Longest gap^c^ (minimum)	4.97 (3.80); 1.27 to 16.85	2.42 (1.77); 0.55 to 10
Number of common measurements^d^ (1000s)	23.54 (2.90); 17.21 to 30.35	21.81 (3.14); 13 to 27.23
*R* ^2e^	0.89 (0.15); 0.03 to 0.96	0.90 (0.06); 0.73 to 0.97
MAE^f^	0.18 (0.04); 0.10 to 0.28	0.17 (0.04); 0.10 to 0.24
Bias	−0.07 (0.02); −0.14 to −0.04	−0.07 (0.02); −0.12 to −0.04
LoA^g^—low	−0.94 (0.35); −2.19 to −0.43	−0.83 (0.28); −1.57 to −0.43
LoA—high	0.80 (0.32); 0.32 to 1.90	0.69 (0.25); 0.32 to 1.36

^a^Number of instantaneous respiratory rate measurements in 1000s.

^b^Percentage of the time Somnofy provided respiratory rate measurements.

^c^Highest number of minutes between 2 Somnofy measurements per night.

^d^Number of times both Somnofy and noise-filtered respiratory inductance plethysmography provided measurement.

^e^*R^2^*: coefficient of determination.

^f^MAE: mean absolute error.

^g^Bland-Altman 95% limits of agreement, calculated as bias – 1.96 × SD to bias + 1.96 × SD.

**Table 4:** Results for instantaneous respiratory rate for Somnofy-defined sleep stages.

Parameters	Wake	Light	Deep	Rapid eye movement
Number of measurements^a^	141,320	539,229	195,462	178,862
RIP^b^—average respiratory rate (RPM^c^)	16.9	15.3	15.7	16.1
Coverage^d^ (%)	47.1	91.3	98.1	75.3
Number of common measurements^e^	62,981	485,315	189,369	132,109
MAE^f^	0.33	0.15	0.11	0.30
Bias	−0.12	−0.06	−0.05	−0.12
LoA^g^—low	−1.97	−0.66	−0.38	−1.67
LoA—high	1.72	0.55	0.28	1.42

^a^Number of instantaneous respiratory rate measurements.

^b^RIP: respiratory inductance plethysmography.

^c^RPM: respirations per minute.

^d^Percentage of the time Somnofy provided respiratory rate measurements.

^e^Number of times both Somnofy and noise-filtered RIP provided measurement.

^f^MAE: mean absolute error.

^g^Bland-Altman 95% limits of agreement, adjusted for repeated measurements and calculated as bias – 1.96 × SD to bias + 1.96 × SD.

### Nightly Average RR

**Figure 3:**
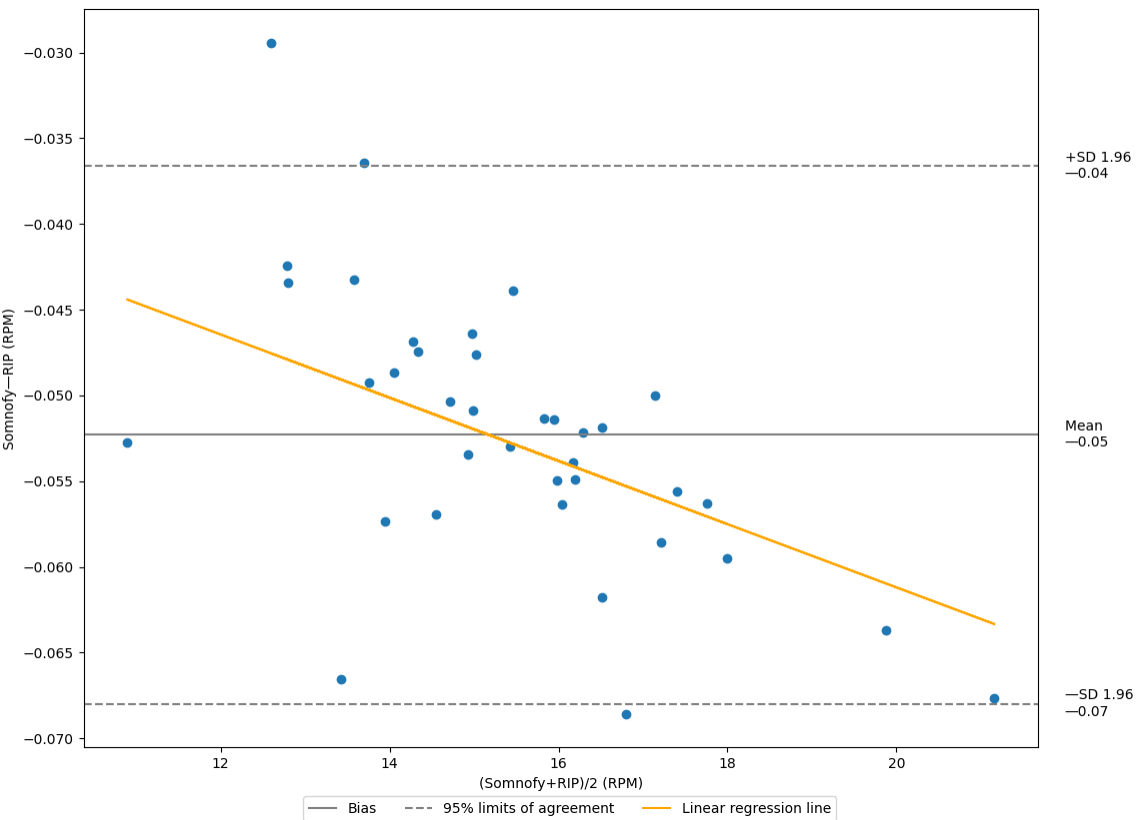
Bland-Altman analysis for nightly filtered respiratory rates. The y-axis displays the disagreement between Somnofy and respiratory inductance plethysmography (RIP; N=37), whereas the x-axis displays the average of Somnofy and RIP measurements. All values are presented as respirations per minute (RPM). The nightly averages are visualized as blue dots.

For the nightly average RRs, MAE was 0.052 (SD 0.008) RPM. Figure 3 displays the Bland-Altman plot for these averages. The Bland-Altman limits of agreement show that 95% of the nightly averages are expected to have a disagreement with RIP between −0.07 and −0.04 RPM. As indicated by the orange line (slope=−0.0018; *R*^2^=0.343), there seems to be a trend, where Somnofy underestimates more for high RRs. This trend is a similar to that for the instantaneous RR measurements shown in Figure 2.

### Night-to-Night Consistency of Nightly Average RR

**Figure 4:**
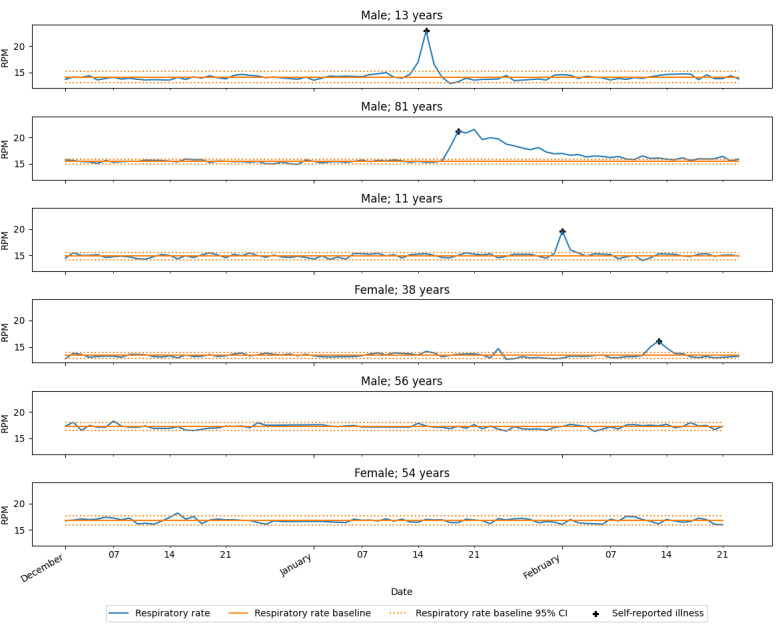
Nightly filtered average respiratory rates over 3 months. A total of 6 individual participants are labeled with sex and age above the corresponding graph. The y-axis displays the average filtered respiratory rates as respirations per minute (RPM), whereas the x-axis displays the date at wake up. Self-reported illness is tagged on the first peak of the respiratory rate during the illness. All nights substantially outside the CI were related to the self-reported illnesses.

The results from the longitudinal pilot study are shown in Figure 4. The RRs were fairly consistent from night to night for all participants (6/6, 100%), and most values were within the 95% CIs around the baselines. Moreover, periods with self-reported illness substantially deviated from their respective baselines. In total, 67% (4/6) of the participants reported 1 illness each. The participants who were aged 13, 11, and 38 years, respectively, reported a cold, and the participant aged 81 years reported an infection that was treated with antibiotics.

### Other Analyses

In this study, the null hypothesis that MAE and coverage were independent of age (MAE: *P*=.97 and coverage: *P*=.63), sex (MAE: *P*=.28 and coverage: *P*=.73), and BMI (MAE: *P*=.99 and coverage: *P*=.43) could not be rejected. In contrast, for sleeping position and sensor location, the null hypothesis was rejected. Some combinations, all including supine sleeping position, showed statistically significantly higher MAE (mean 0.045 RPM, SD 0.01) and statistically significantly lower coverage (mean 5.0%, SD 0.9%) than other radar or sleeping positions. The mean difference and *P* values for all the significantly different combinations are shown in Table 5.

**Table 5:** Statistically significant differences for sleeping position and sensor location^a^.

Significantly different combinations	MAE^b^ (RPM^c^)			Coverage^d^ (%)	
	Mean difference	*P* value	Mean difference		*P* value
Supine nightstand—left wall	0.048	<.001	−6.21		<.001
Supine nightstand—right wall	0.046	<.001	−5.99		<.001
Supine nightstand—left nightstand	0.052	.002	−4.78		<.001
Supine nightstand—right nightstand	0.042	<.001	−4.17		.002
Supine nightstand—prone wall	0.053	.02	N/A^e^		N/A
Supine wall—right wall	0.027	.04	−4.15		.002
Supine wall—left wall	N/A	N/A	−4.37		.001

^a^The table shows combinations of sleeping position (right, left, supine, and prone) and sensor location (nightstand and wall) that were statistically significantly different using Tukey method on a linear mixed effects model (33/37, 89%).

^b^MAE: mean absolute error.

^c^RPM: respirations per minute.

^d^Percentage of the time Somnofy provided respiratory rate measurements.

^e^N/A: not applicable.

## Discussion

### Principal Findings

This study demonstrated that Somnofy can accurately detect instantaneous RRs during time in bed for healthy adults. On average, Somnofy was able to measure RR 84% (SD 6%) of the time with MAE of 0.18 (SD 0.04) RPM. The Bland-Altman 95% limits of agreement ranged from −0.99 to 0.85 RPM. The accuracy and coverage varied significantly according to the sleep stage, where deep sleep (MAE=0.11; coverage=98%) was the most accurate, followed by light sleep (MAE=0.15; coverage=91%), REM sleep (MAE=0.30; coverage=75%), and wake (MAE=0.33; coverage=47%). For filtered nightly averages, the measurements from RIP and Somnofy were almost identical with Bland-Altman 95% limits of agreement, ranging from −0.07 to −0.04 RPM. Overall, Somnofy tended to slightly underestimate RR. Results were independent of age, BMI, and sex but were slightly worse for supine sleeping position.

The longitudinal part of the study showed that the nightly RRs seem fairly consistent from night to night. Most nightly averages were within the 95% CIs of the personalized baselines. Moreover, the CIs were smaller than, for example, the normal effect of 1-degree increase in body temperature on RR (eg, associated with fever) [5,6]. All substantial deviations coincided with self-reported illness, which were expected to increase the RR. The small night-to-night variations can be caused by other factors that affect RR, such as increased RRs after intense workouts [12] and RRs varying with emotions or anxiety [13] and owing to menstrual cycle [14]. A large study investigating these factors is necessary to understand the usefulness of investigating small deviations from baseline.

The coverage and accuracy varied substantially according to the sleep stage. It is probably easier for Somnofy to measure RR during light and deep sleep, as RR during these periods is more stable than that during wake or REM sleep. Coverage was particularly low during wake, when more movement likely resulted in more noise. Somnofy takes advantage of this by using values only from light and deep sleep to calculate the nightly average, during which the accuracy is higher. This has the additional benefit that the average is independent of the amount of wake and REM sleep during the night, during which RR varies more and tends to be higher. Thus, using values only from light and deep sleep should also improve the night-to-night consistency.

On average, 5.8% (SD 1.9%) of the PSG data had to be filtered for noise. Another reference device or signal processing technique could have been used instead of RIP with STFT. Unfortunately, there is no gold standard for longitudinal measurements of RR, and changing the reference is unlikely to affect the results significantly. RIP was used in one study [23], and RIP and STFT were used in another study [27]. One study used a nasal flow sensor, but had to remove 10 out of 40 nights owing to missing or unusable flow data [24]. Another study used both flow and effort signals with a peak detection algorithm, but based on the number of epochs they analyzed divided by the average time in bed in their study, they removed approximately twice as much noise as removed in this study [25].

Compared with wearables, radar technology has the benefit that nothing must be attached to the body, which can negatively affect sleep. Wearables also must be charged, and the user must remember to wear the device in bed. According to Fitbit, only half of its users wear their armbands at night [42], indicating that compliance can be a problem. Furthermore, neither wearables nor under-the-mattress sensors have been shown to measure sleep as reliably as Somnofy [37], information that can be used for more consistent nightly averages.

As Somnofy measures RRs from a distance, it was especially interesting to analyze the results across sleeping positions and sensor locations. All significant differences were found for supine sleeping position, indicating that this position may be more difficult to assess. For the nightstand sensor, the supine position may be more difficult, because the respiration movement is mainly perpendicular to the transmitted radar signals. This is also true for the prone position, but in this position, some of the respiration movements may be pushed in different directions by the bed. The wall sensor may have difficulties in assessing the supine position owing to more movement. Here, the body is free to move, and the sensor has good view of the movements. Interestingly, Somnofy seems to measure RR equally well when the chest or the back is aimed toward the sensor. In addition, there was no statistically significant difference according to age, sex, or BMI, which are factors that can affect both how respiration is visible on the body surface and actual RRs. However, the study had few participants with high BMI; therefore, this should be investigated further.

Previous studies have reported that active measurements of RRs can be imprecise if the user is aware of being measured, and therefore, consciously affects breathing [43]. As Somnofy measures RRs from a distance, it should be possible to do so without affecting the user. Moreover, measuring during sleep enforces measurements to occur during rest. Noncontact measurements of RRs should also have other benefits such as user-friendliness and less administration.

### Comparison With Previous Studies

Few commercially available technologies suitable for longitudinal studies have been validated for RR measurements. Comparison between studies is always difficult, because the sleep data, reference device or signal processing, and performance metrics are different. However, to the best of our knowledge, the results of this study are significantly better than those of other technologies [23-25,44-46] including radar technology [27,29]. The validated measurements are also more instantaneous, as previous studies have averaged RRs over epochs of data. Moreover, previous studies of noncontact RR measurements during sleep have not investigated the effect of all the factors that can affect the results, such as sleep stage, age, BMI, and sleeping position [23-25,27,46]. This is also the first study that explicitly analyses coverage and measures gaps in continuous RR measurements during sleep using radar technology.

To the best of our knowledge, the accuracy in this study is also significantly higher than that of the measuring methods used in hospitals, such as manual counting of breaths [47] and chest patches [19]. However, these studies were performed on different populations and in different settings, which could have made measurements more difficult.

Previous studies have not validated filtered nightly average, as proposed in this study. This filtered average was substantially more accurate than the standard nightly averages reported in other studies [24,25]. In theory, it should also be more suitable for longitudinal studies, as the night-to-night variability should be lower.

No other study has analyzed whether anomaly detection from personalized baselines is a sound application of longitudinal RR monitoring. For the approach to be sensible, RRs need to be sufficiently consistent from night to night and the measurement error needs to be sufficiently small, depending on the application. However, a pilot study investigated a specific use case for patients with COPD [48]. Their intention was to detect exacerbation of COPD by comparing the median RR of one night with those of the previous nights. They concluded that RR can be obtained using radar technology and that RR may be an indicator of change in clinical status. Another study found that the use of both instantaneous and previous RRs improved precision when detecting clinical deterioration [49].

### Limitations

The study investigated measurements only during rest, and the results cannot be automatically applied to general situations where the individual is awake. Furthermore, the study was limited to healthy adults. More studies are necessary to validate Somnofy for people with different illnesses, children, and older adults.

The second part of the study included few participants. A large population should be analyzed to investigate the night-to-night consistency of average nocturnal RRs in a general population. Moreover, the participants’ illnesses were self-reported. No physicians were consulted for diagnosis, and body temperatures were not measured. Further studies should investigate which types of disease can be detected with this technology and how early in the development of these diseases the RR changes.

This study analyzed only RR. Although RR can be valuable to measure longitudinally, more value can be added by measuring other biomarkers such as temperature, blood pressure, and heart rate simultaneously. Heart rate is often measured using comparable technology [23-25], and radar technology has previously also been validated for measuring heart rate during sleep [27]. Heart rate measurement was not available from Somnofy at the time of this study.

### Conclusions

This study shows that Somnofy accurately measures RR during sleep in healthy adults. To the best of our knowledge, Somnofy has higher precision than any other noncontact device suitable for longitudinal monitoring, especially for nightly averages. Moreover, measuring RRs during sleep seems to be a sound option for consistent longitudinal measurements. Several events that affect the RR should be detectable as deviations from personalized nocturnal baselines, making the device suitable for a broad range of applications. Further studies are necessary to validate the use of Somnofy for children and older adults or to use this device in clinical settings.

## Appendix

Multimedia Appendix 1 Instantaneous respiratory rates across PSG-defined sleep stages.

## Abbreviations

COPD: chronic obstructive pulmonary disease
FT: Fourier transform
MAE: mean absolute error
PSG: polysomnography
REM: rapid eye movement
RIP: respiratory inductance plethysmography
RPM: respirations per minute
RR: respiratory rate
STFT: short-time Fourier transform
