# Supplementary material for: Noncontact Longitudinal Respiratory Rate Measurements in Healthy Adults Using Radar-Based Sleep Monitor (Somnofy): Validation Study
Source: JMIR Biomed Eng. 2022 Aug 12;7(2):e36618. doi: 10.2196/36618 (PMC11041471; doi:10.2196/36618)
Supplement: Multimedia Appendix 1 [file biomedeng_v7i2e36618_app1.docx]

Table A1. Results for instantaneous respiratory rate for different PSG-defined sleep stages^a^

|  | Wake | Light | Deep | REM |
| --- | --- | --- | --- | --- |
|  |  |  |  |  |
| N | 151 150 | 599 894 | 131 912 | 171 827 |
| RIP average respiratory rate (RPM) | 16.5 | 15.4 | 15.8 | 16.0 |
| Coverage (%) | 48.5 | 91.9 | 97.7 | 76.5 |
| N common (1000s) | 69 877 | 543 738 | 127 131 | 129 028 |
| MAE | 0.34 | 0.15 | 0.11 | 0.31 |
| Bias | -0.12 | -0.06 | -0.05 | -0.12 |
| LoA- low | -2.07 | -0.60 | -0.39 | -1.61 |
| LoA - high | 1.82 | 0.49 | 0.29 | 1.37 |

^a^Results across sleep stages. N = number of instantaneous respiratory rate measurements, RIP = respiratory inductance plethysmography, RPM = respirations per minute, coverage = percent of the time Somnofy provided respiratory rate measurements, N common = number of times both Somnofy and noise filtered RIP provided measurement, MAE = mean absolute error, LoA = Bland Altman 95% limits of agreement adjusted for repeated measurements calculated as bias ± 1.96 SD.
